# Complete nucleotide sequence of a novel mycovirus from *Trichoderma harzianum* in China

**DOI:** 10.1007/s00705-019-04145-9

**Published:** 2019-02-12

**Authors:** Chenchen Liu, Mei Li, Estifanos Tsegaye Redda, Jie Mei, Jiantai Zhang, Santiago F. Elena, Beilei Wu, Xiliang Jiang

**Affiliations:** 1 Institute of Plant Protection, Chinese Academy of Agricultural Sciences, No. 2 West Yuanmingyuan Rd., Haidian District, Beijing, 100193 China; 2 Instituto de Biología Integrativa de Sistemas (CSIC-Universitat de València), Parc Cientific UV, Catedrático Agustín Escardino 9, 46980 Paterna, Valencia Spain; 3 The Santa Fe Institute, 1399 Hyde Park Road, Santa Fe, NM 87501 USA

## Abstract

A new mycovirus was identified in *Trichoderma harzianum* strain 137 isolated in Xinjiang province, China. The whole genome sequence of the mycovirus was determined by metagenomic sequencing, RT-PCR, and RACE cloning. The mycovirus contained two genomic segments. The first segment was 2088 bp long and contained a single ORF (ORF1) encoding the RNA-dependent RNA polymerase (RdRP) (72.26 kDa). The second segment was 1634 bp long and also contained a single ORF (ORF2) encoding a hypothetical protein of 37.472 kDa. We named this novel mycovirus “Trichoderma harzianum bipartite mycovirus 1” (ThBMV1). Phylogenetic analysis showed that ThBMV1 clusters with other unclassified dsRNA mycoviruses.

**Electronic supplementary material:**

The online version of this article (10.1007/s00705-019-04145-9) contains supplementary material, which is available to authorized users.

## Introduction

At present, only three mycoviruses infecting *Trichoderma* have been described [1–3]. In 2009, Jom-in and Akarapisan provided the first description of two mycoviruses, with sizes of 0.7 kb and 1.1 kb, respectively, isolated from *Trichoderma* [1]. Later, Yun et al. suggested that mycoviruses from *Lentinula edodes* were widespread in Korea and isolated 32 different dsRNA-containing viruses from 315 strains of *Trichoderma* spp. [2]. More recently, Lee et al. isolated an unclassified mycovirus from *Trichoderma atroviride*, naming it “Trichoderma atroviride mycovirus 1” (TaMV 1) [3]. To expand the list of mycoviruses infecting *Trichoderma* spp., we have isolated 152 *Trichoderma* spp. from soil samples obtained from the Chinese provinces of Xinjiang, Inner Mongolia, Heilongjiang, and Jilin. These isolates were classified at the species level based on morphological properties and molecular data (sequencing of the ITS region, the translation elongation factor 1-α (*tef1-α*) gene, and the RNA polymerase subunit II (*rpb*2) gene). In an effort to identify new *Trichoderma* viruses, all of these isolates were screened for the presence of mycoviruses (Supplemental Table 1).

## Identification of a novel *Trichoderma* mycovirus

**Fig. 1:**
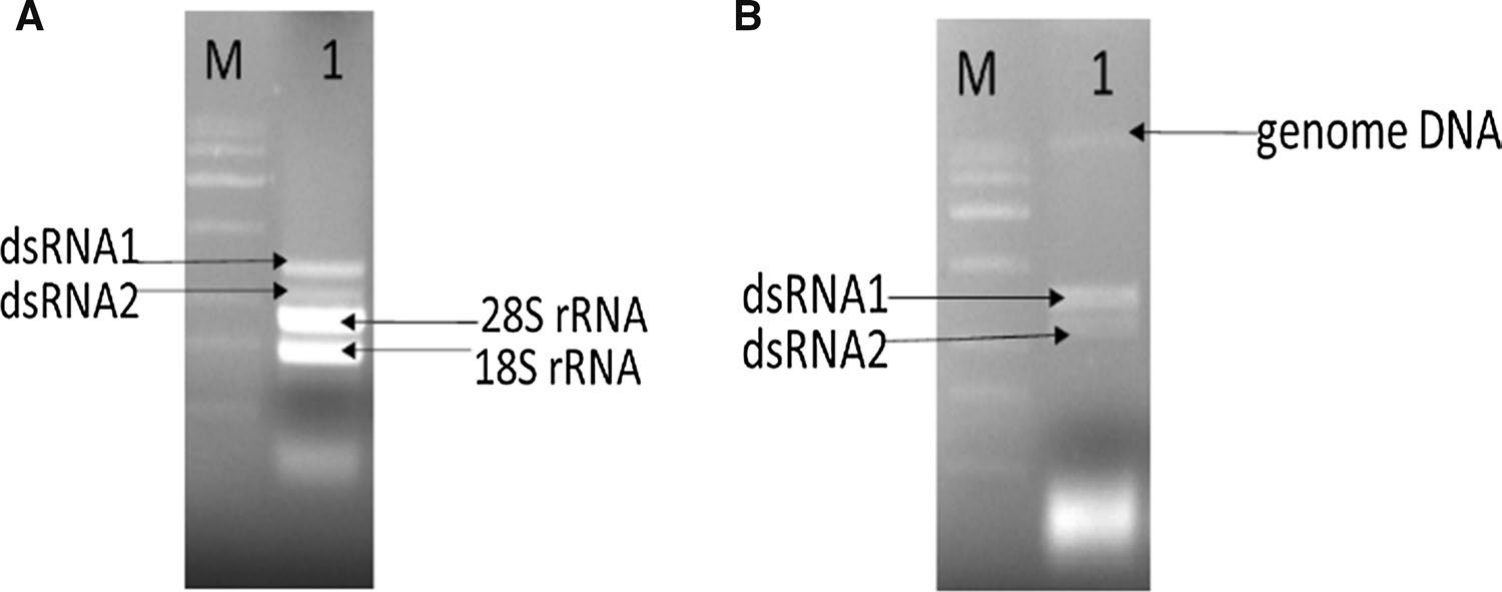
Detection of dsRNA from *Trichoderma harzianum* strain 137 by digestion with DNase Ι and S1 nuclease, successively. (**A**) The dsRNA sample was treated with DNase Ι and electrophoresed in a 1.5% agarose gel and detected on a UV transilluminator. (**B**) dsRNA sample was treated with S1 nuclease and electrophoresed in a 1.5% agarose gel and detected on a UV transilluminator

After extraction of dsRNA from mycelia [4], it was possible to visualize two nucleic acid segments with 2 and 1.5 kb, respectively, in strain 137. After extraction, the dsRNA was treated with DNase I and S1 nuclease to confirm the nature of the nucleic acid (Fig. 1A and B). dsRNAs were sent to Shanghai Biotechnology Corporation for sequencing using Illumina HiSeq2500 equipment. Raw data were analyzed by the company, including data preprocessing, sequence assembly, database annotation, gene quantification, species classification, and species abundance analysis. The resulting contigs were annotated using RefSeq by comparing them with the NCBI non-redundant (NR) protein database using BLASTx (https://blast.ncbi.nlm.nih.gov/Blast.cgi). Two contigs of about 2 and 1.5 kb were identified, which are hereafter referred to as RNA1 and RNA2, respectively. Using the EMBOSS-NEEDLE program (https://www.ebi.ac.uk/Tools/emboss/), we found that RNA 1 showed the highest nucleotide sequence similarity (65.7% identity) to Cryphonectria parasitica bipartite mycovirus 1, while RNA 2 showed the highest nucleotide sequence similarity (55.5% identity) to Penicillium aurantiogriseum bipartite virus 1 (Supplemental Table 2). Based on the contig sequences, RT-PCR primers specific for dsRNA1 (forward, AATCGCGCTTACCCGTAACC; reverse, AGTAACTTCGGCACCATCGTCC) and dsRNA2 (forward, AACCACCTCCTCAATCCCTTCC; reverse, TGGGTGACAGTTTTGAAGAGTTGCAGCG) were designed. The RT-PCR assays were repeated to confirm the presence of the virus, and the resulting fragments were sequenced. The resulting sequences consisted of contigs of 1116 and 359 nt (Supplemental Fig. 2). The 5′ and 3′ ends of the bipartite genome were determined using classical RACE methods [5, 6]. The complete genome sequence was assembled using DNAMAN software and deposited in the GenBank database under the accession numbers MH536648 and MH536649.

## Genomic properties

The larger segment of 2088 bp was named dsRNA1, and the smaller segment of 1634 bp was named dsRNA2. Both included a poly(A) tail. The G+C content of the dsRNA1 segment was 50.0%, and for dsRNA2 it was 50.3%. Possible ORFs were found using the NCBI ORF finder tool (https://www.ncbi.nlm.nih.gov/orffinder/). Each dsRNAs contained a single ORF in the positive-sense strand. The 5′ UTR of dsRNA1 was 78 bp long, and the 3′ UTR was 114 bp long; they had no hairpin structures in the UTR regions. The 5′ UTR of dsRNA2 was 105 bp long, while the 3′ UTR was 584 bp long; the structure of UTR regions was the same as for dsRNA1. The 5′ UTRs of dsRNA1 and dsRNA2 had the same conserved element (CUGAGUUAACAAGCCACUGUUUUACUCUCGU), which is necessary for virus replication. A BLASTX search for homologues of ORF1 (nt 79-1974) showed that ORF1 most likely encodes an RNA-dependent RNA polymerase (RdRP) of 631 amino acids with a molecular weight of 72.3 kDa. The C-terminal domain and catalytic palm subdomain were detected in the regions encompassing amino acids 300-448 and 326-454, respectively, using InterPro (http://www.ebi.ac.uk/interpro). Likewise, ORF2 (nt 106-1050) was predicted to encode a hypothetical protein of 314 amino acids with a molecular weight of 37.5 kDa with similarity to other proteins of unknown function or coat proteins from unclassified dsRNA mycoviruses and members of the family *Partitiviridae* (Supplementary Table 3).

**Fig. 2:**
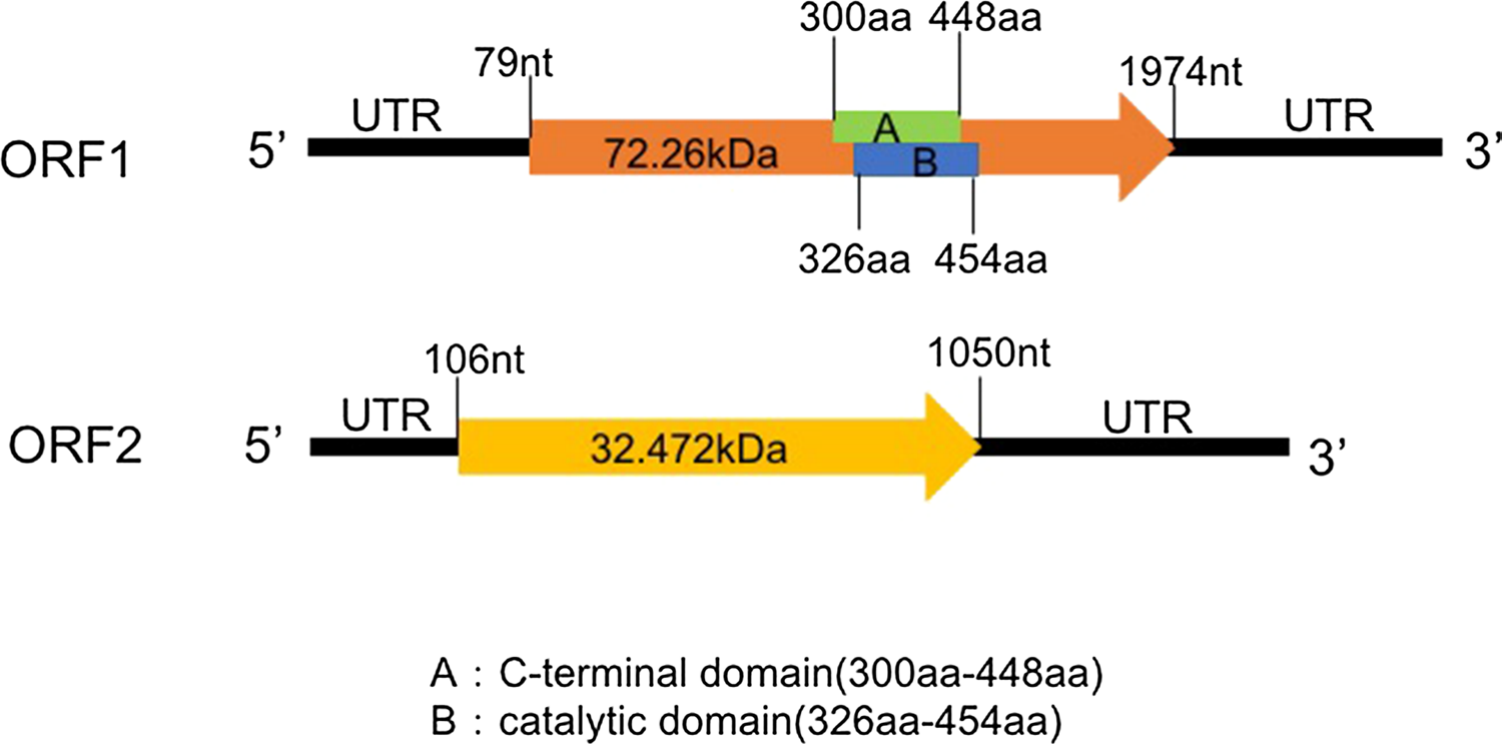
Schematic representation of the genomic organization of ThBMV1. The dsRNA1 genome contains one ORF. ORF 1 encodes the RNA-dependent RNA polymerase (RdRp). In the RdRp, the C-terminal domain and catalytic domain are located in the regions encompassing amino acids 300-448 and 326-454, respectively. The dsRNA2 genome contains one ORF, ORF 2, which encodes a hypothetical protein. The black lines indicate 5′ and 3′ UTRs

**Fig. 3:**
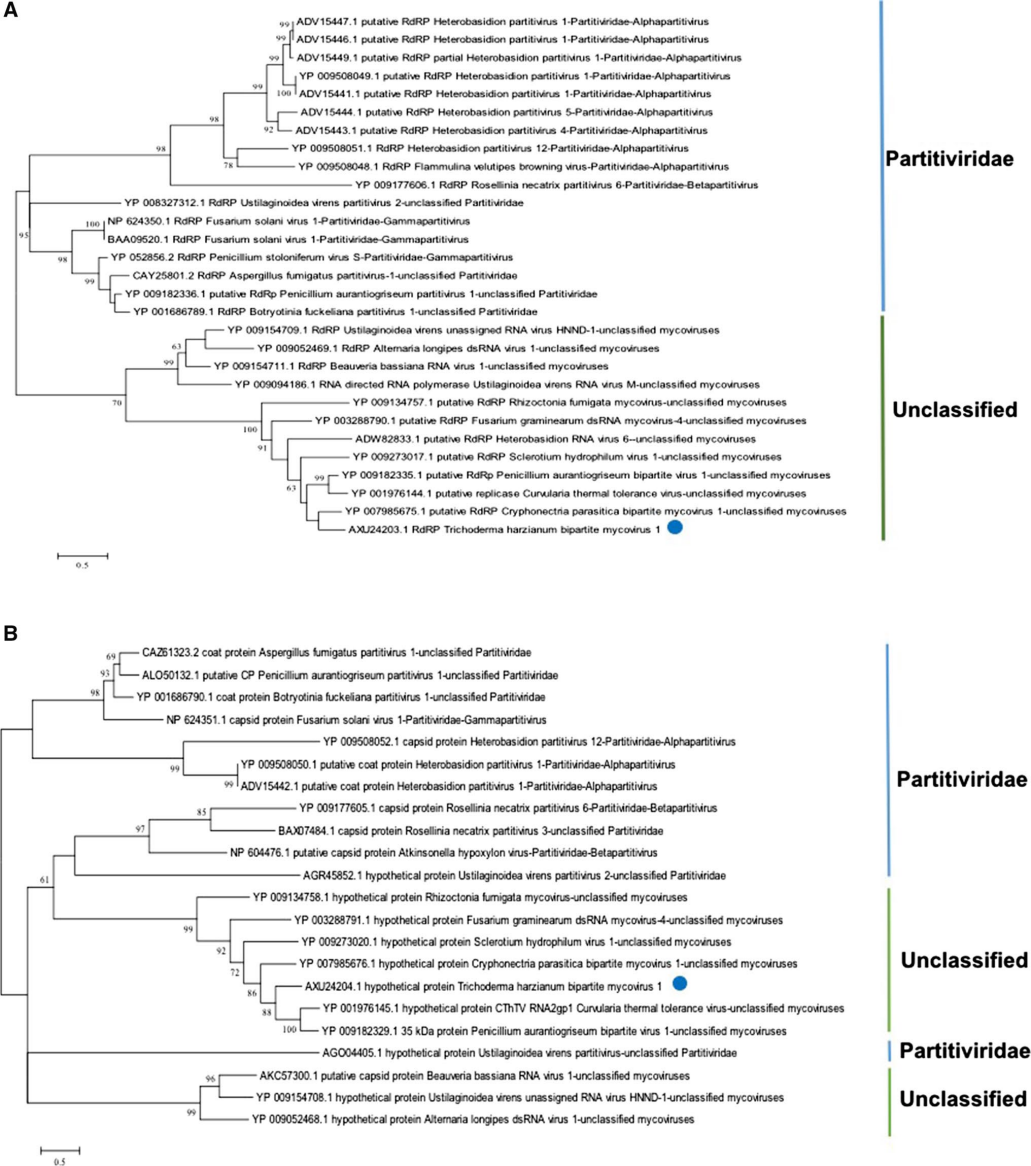
Maximum-likelihood phylogenetic trees inferred from the amino acid sequences of ORF1 and ORF2. Numbers at the nodes represent the statistical support for each cluster (based on 1000 bootstrap replicates). (**A**) Phylogenetic tree for ORF1 (encoding RdRP), using LG+G+I as the best model of amino acid substitution. (**B**) Phylogenetic tree for ORF2 (encoding the hypothetical protein), using WAG+G+I as the best model of amino acid substitution

Phylogenetic trees were constructed using the amino acid sequences of RdRps from 29 fungal viruses and hypothetical proteins or coat proteins from 22 mycoviruses. The protein sequences for these viruses were retrieved from the GenBank database and included four bipartite mycoviruses: Cryphonectria parasitica bipartite mycovirus 1, Penicillium aurantiogriseum bipartite virus 1 [7], Heterobasidion RNA virus 6 [8], and Curvularia thermal tolerance virus [9]. Maximum-likelihood phylogenetic trees were constructed using MEGA 7.0 software [10] (Supplemental Fig. 3 and Supplemental Tables 4, 5 and 6) and using the best amino acid substitution model (LG + G for the RdRP and WAG + G + I for the hypothetical or coat protein), and their statistical significance was evaluated by bootstrap (based on 1000 pseudoreplicates). In both phylogenies, the novel mycovirus clustered with a group of unclassified mycoviruses (Fig. 2A and B; Supplemental Tables 4, 5 and 6). For the sake of completeness, an alignment resulting from concatenating the aligned RdRP and hypothetical protein sequences was also used to construct a single maximum-likelihood phylogenetic tree using the WAG+G+I model (Supplemental Fig. 2). The results were identical to those obtained using each protein independently. Based on the above results, we concluded that the mycovirus isolated from *T. harzianum* isolate 137 was a novel unclassified mycovirus. We have tentatively named it “Trichoderma harzianum bipartite mycovirus 1” (ThBMV1) (Fig. 3).

## Electronic supplementary material

Below is the link to the electronic supplementary material.

Supplementary material 1 (DOCX 291 kb)
