# Supplementary material for: Complete nucleotide sequence of a novel mycovirus from Trichoderma harzianum in China
Source: Arch Virol. 2019 Feb 12;164(4):1213–6. doi: 10.1007/s00705-019-04145-9 (PMC6420475; doi:10.1007/s00705-019-04145-9)

Supplemental Table 1. The information of *Trichoderma* strains collected from Xinjiang, Inner Mongolia, Jilin and Heilongjiang provinces of China

| The order of the isolates | Strain Number | Strain Name | Region |
| --- | --- | --- | --- |
| 1 | CTCCSJ-G-QT40002 | *Hypocrea lixii/Trichoderma harzianum* | Xinjiang |
| 2 | CTCCSJ-G-QT40003 | *Hypocrea lixii/Trichoderma harzianum* | Xinjiang |
| 3 | CTCCSJ-G-QT40004 | *Hypocrea lixii/Trichoderma harzianum* | Xinjiang |
| 4 | CTCCSJ-G-HB40005 | *Trichoderma viridescens* | Xinjiang |
| 5 | CTCCSJ-F-ZY40007 | *Hypocrea schweinitzii/Trichoderma citrinoviride* | Xinjiang |
| 6 | CTCCSJ-F-ZY40008 | *Hypocrea schweinitzii/Trichoderma citrinoviride* | Xinjiang |
| 7 | CTCCSJ-F-ZY40009 | *Hypocrea schweinitzii/Trichoderma citrinoviride* | Xinjiang |
| 8 | CTCCSJ-G-JK40012 | *Hypocrea lixii/Trichoderma harzianum* | Xinjiang |
| 9 | CTCCSJ-G-HB40015 | *Hypocrea lixii/Trichoderma harzianum* | Xinjiang |
| 10 | CTCCSJ-G-HB40017 | *Trichoderma harzianum* | Xinjiang |
| 11 | CTCCSJ-G-HB40019 | *Trichoderma afroharzianum* | Xinjiang |
| 12 | CTCCSJ-F-ZY40020 | Hypocrea schweinitzii/Trichoderma citrinoviride | Xinjiang |
| 13 | CTCCSJ-F-KY40021 | Hypocrea schweinitzii/Trichoderma citrinoviride | Xinjiang |
| 14 | CTCCSJ-F-ZY40022 | Hypocrea schweinitzii/Trichoderma citrinoviride | Xinjiang |
| 15 | CTCCSJ-F-ZY40023 | Hypocrea schweinitzii/Trichoderma citrinoviride | Xinjiang |
| 16 | CTCCSJ-G-HB40030 | *Hypocrea pseudoharzianum* | Inner Mongolia |
| 17 | CTCCSJ-G-HB40031 | *Hypocrea pseudoharzianum* | Inner Mongolia |
| 18 | CTCCSJ-G-HB40032 | Hypocrea schweinitzii/Trichoderma citrinoviride | Inner Mongolia |
| 19 | CTCCSJ-G-HB40038 | Hypocrea schweinitzii/Trichoderma citrinoviride | Inner Mongolia |
| 20 | CTCCSJ-G-HB40040 | Trichoderma longibrachiatum | Inner Mongolia |
| 21 | CTCCSJ-G-HB40046 | Hypocrea schweinitzii/Trichoderma citrinoviride | Inner Mongolia |
| 22 | CTCCSJ-F-ZY40048 | *Trichoderma afroharzianum* | Xinjiang |
| 23 | CTCCSJ-G-HB40050 | *Trichoderma afroharzianum* | Inner Mongolia |
| 24 | CTCCSJ-F-KY40053 | *Trichoderma afroharzianum* | Inner Mongolia |
| 25 | CTCCSJ-G-HB40055 | *Trichoderma afroharzianum* | Xinjiang |
| 26 | CTCCSJ-G-HB40057 | *Trichoderma longibrachiatum* | Inner Mongolia |
| 27 | CTCCSJ-G-HB40061 | *Trichoderma afroharzianum* | Inner Mongolia |
| 28 | CTCCSJ-G-HB40065 | *Trichoderma afroharzianum* | Inner Mongolia |
| 29 | CTCCSJ-G-JK40067 | *Trichoderma afroharzianum* | Xinjiang |
| 30 | CTCCSJ-G-HB40091 | *Hypocrea pseudoharzianum* | Inner Mongolia |
| 31 | CTCCSJ-G-QT40119 | *Trichoderma viridescens* | Jilin |
| 32 | CTCCSJ-F-ZY40121 | *Trichoderma koningiopsis* | Jilin |
| 33 | CTCCSJ-G-HB40124 | *unidentified* | Xinjiang |
| 34 | CTCCSJ-G-HB40137(137) | *Trichoderma harzianum* | Xinjiang |
| 35 | CTCCSJ-G-HB40142 | *Trichoderma afroharzianum* | Xinjiang |
| 36 | CTCCSJ-G-QT40148 | *Trichoderma longibrachiatum* | Inner Mongolia |
| 37 | CTCCSJ-G-QT40151 | *Trichoderma longibrachiatum* | Inner Mongolia |
| 38 | CTCCSJ-F-KY40153 | *Trichoderma longibrachiatum* | Inner Mongolia |
| 39 | CTCCSJ-G-QT40154 | *Trichoderma longibrachiatum* | Inner Mongolia |
| 40 | CTCCSJ-F-KY40155 | *Trichoderma longibrachiatum* | Inner Mongolia |
| 41 | CTCCSJ-G-HB40167 | *Trichoderma longibrachiatum* | Inner Mongolia |
| 42 | CTCCSJ-G-QT40168 | *Trichoderma longibrachiatum* | Inner Mongolia |
| 43 | CTCCSJ-G-HB40188 | *Trichoderma longibrachiatum* | Xinjiang |
| 44 | CTCCSJ-G-HB40189 | *Trichoderma longibrachiatum* | Xinjiang |
| 45 | CTCCSJ-G-HB40190 | *Trichoderma longibrachiatum* | Xinjiang |
| 46 | CTCCSJ-G-QT40192 | *Trichoderma longibrachiatum* | Xinjiang |
| 47 | CTCCSJ-G-QT40194 | *Trichoderma longibrachiatum* | Inner Mongolia |
| 48 | CTCCSJ-G-HB40218 | *Trichoderma longibrachiatum* | Inner Mongolia |
| 49 | CTCCSJ-G-QT40222 | *Trichoderma longibrachiatum* | Inner Mongolia |
| 50 | CTCCSJ-G-QT40223 | *Trichoderma longibrachiatum* | Inner Mongolia |
| 51 | CTCCSJ-G-HB40226 | *Trichoderma longibrachiatum* | Xinjiang |
| 52 | CTCCSJ-G-JK40241 | *Trichoderma longibrachiatum* | Xinjiang |
| 53 | CTCCSJ-G-JK40242 | *Trichoderma longibrachiatum* | Xinjiang |
| 54 | CTCCSJ-G-HB40244 | *Trichoderma afroharzianum* | Inner Mongolia |
| 55 | CTCCSJ-F-ZY40245 | *Trichoderma afroharzianum* | Inner Mongolia |
| 56 | CTCCSJ-F-ZY40246 | *Trichoderma afroharzianum* | Xinjiang |
| 57 | CTCCSJ-F-ZY40248 | *Trichoderma longibrachiatum* | Xinjiang |
| 58 | CTCCSJ-G-QT40252 | *Trichoderma afroharzianum* | Inner Mongolia |
| 59 | CTCCSJ-G-JK40253 | *Trichoderma afroharzianum* | Xinjiang |
| 60 | CTCCSJ-G-HB40257 | *Trichoderma longibrachiatum* | Inner Mongolia |
| 61 | CTCCSJ-G-HB40263 | *Trichoderma longibrachiatum* | Inner Mongolia |
| 62 | CTCCSJ-F-ZY40265 | *Trichoderma longibrachiatum* | Xinjiang |
| 63 | CTCCSJ-G-HB40270 | *Trichoderma longibrachiatum* | Xinjiang |
| 64 | CTCCSJ-G-QT40271 | *Trichoderma longibrachiatum* | Inner Mongolia |
| 65 | CTCCSJ-G-QT40274 | *Trichoderma longibrachiatum* | Inner Mongolia |
| 66 | CTCCSJ-G-HB40275 | *Trichoderma longibrachiatum* | Inner Mongolia |
| 67 | CTCCSJ-G-HB40276 | *Trichoderma longibrachiatum* | Xinjiang |
| 68 | CTCCSJ-F-KY40280 | *Trichoderma longibrachiatum* | Inner Mongolia |
| 69 | CTCCSJ-G-QT40283 | *Trichoderma longibrachiatum* | Inner Mongolia |
| 70 | CTCCSJ-G-HB40284 | *Trichoderma afroharzianum* | Inner Mongolia |
| 71 | CTCCSJ-G-JK40288 | *Trichoderma afroharzianum* | Xinjiang |
| 72 | CTCCSJ-G-QT40290 | *Trichoderma afroharzianum* | Inner Mongolia |
| 73 | CTCCSJ-G-JK40292 | *Trichoderma afroharzianum* | Xinjiang |
| 74 | CTCCSJ-F-ZY40300 | *Trichoderma afroharzianum* | Inner Mongolia |
| 75 | CTCCSJ-G-DK40302 | *Trichoderma longibrachiatum* | Inner Mongolia |
| 76 | CTCCSJ-G-QT40303 | *Trichoderma asperelloides* | Xinjiang |
| 77 | CTCCSJ-G-QT40304 | *Trichoderma asperelloides* | Inner Mongolia |
| 78 | CTCCSJ-G-HB40311 | *Trichoderma viridescens* | Heilongjiang |
| 79 | CTCCSJ-G-QT40314 | *Trichoderma koningiopsis* | Inner Mongolia |
| 80 | CTCCSJ-G-QT40315 | *Trichoderma koningiopsis* | Inner Mongolia |
| 81 | CTCCSJ-G-HB40322 | *Trichoderma viridescens* | Xinjiang |
| 82 | CTCCSJ-G-QT40323 | *Trichoderma viridescens* | Jilin |
| 83 | CTCCSJ-G-QT40324 | *Trichoderma koningiopsis* | Inner Mongolia |
| 84 | CTCCSJ-G-HB40325 | *Trichoderma koningiopsis* | Inner Mongolia |
| 85 | CTCCSJ-G-HB40327 | *Trichoderma koningiopsis* | Inner Mongolia |
| 86 | CTCCSJ-G-HB40340 | *Hypocrea lixii/Trichoderma harzianum* | Inner Mongolia |
| 87 | CTCCSJ-F-ZY40342 | *Trichoderma afroharzianum* | Xinjiang |
| 88 | CTCCSJ-F-ZY40354 | *Trichoderma afroharzianum* | Xinjiang |
| 89 | CTCCSJ-G-QT40356 | *Trichoderma afroharzianum* | Inner Mongolia |
| 90 | CTCCSJ-F-ZY40358 | *Trichoderma longibrachiatum* | Jilin |
| 91 | CTCCSJ-G-HB40372 | *Trichoderma longibrachiatum* | Xinjiang |
| 92 | CTCCSJ-G-HB40373 | *Trichoderma longibrachiatum* | Inner Mongolia |
| 93 | CTCCSJ-G-JK40376 | *Hypocrea lixii/Trichoderma harzianum* | Xinjiang |
| 94 | CTCCSJ-G-QT40408 | *Trichoderma longibrachiatum* | Inner Mongolia |
| 95 | CTCCSJ-G-HB40421 | *Hypocrea atroviridis* | Xinjiang |
| 96 | CTCCSJ-G-HB40423 | *Hypocrea koningii/Trichoderma koningii* | Xinjiang |
| 97 | CTCCSJ-G-HB40425 | *Hypocrea atroviridis* | Xinjiang |
| 98 | CTCCSJ-G-HB40429 | *Trichoderma longibrachiatum* | Xinjiang |
| 99 | CTCCSJ-G-HB40430 | *Trichoderma viridescens* | Xinjiang |
| 100 | CTCCSJ-F-ZYB40431 | *Trichoderma viridescens* | Xinjiang |
| 101 | CTCCSJ-G-HB40433 | *Hypocrea lixii/Trichoderma harzianum* | Xinjiang |
| 102 | CTCCSJ-G-QT40435 | *Hypocrea lixii/Trichoderma harzianum* | Xinjiang |
| 103 | CTCCSJ-G-HB40436 | *Hypocrea atroviridis* | Xinjiang |
| 104 | CTCCSJ-F-ZY40439 | *Trichoderma viridescens* | Xinjiang |
| 105 | CTCCSJ-G-HB40440 | *Trichoderma viridescens* | Xinjiang |
| 106 | CTCCSJ-G-HB40441 | *Hypocrea lixii/Trichoderma harzianum* | Xinjiang |
| 107 | CTCCSJ-G-QT40442 | *Hypocrea lixii/Trichoderma harzianum* | Xinjiang |
| 108 | CTCCSJ-G-HB40444 | *Hypocrea lixii/Trichoderma harzianum* | Xinjiang |
| 109 | CTCCSJ-G-QT40447 | *Hypocrea lixii/Trichoderma harzianum* | Xinjiang |
| 110 | CTCCSJ-G-HB40448 | *Hypocrea koningii/Trichoderma koningii* | Xinjiang |
| 111 | CTCCSJ-F-ZY40453 | *Hypocrea koningii/Trichoderma koningii* | Xinjiang |
| 112 | CTCCSJ-G-HB40455 | *Hypocrea lixii/Trichoderma harzianum* | Xinjiang |
| 113 | CTCCSJ-G-HB40456 | *Trichoderma gamsii* | Xinjiang |
| 114 | CTCCSJ-G-HB40461 | *Trichoderma guizhouense* | Inner Mongolia |
| 115 | CTCCSJ-G-HB40462 | *Hypocrea lixii/Trichoderma harzianum* | Inner Mongolia |
| 116 | CTCCSJ-G-QT40476 | *Trichoderma koningiopsis* | Inner Mongolia |
| 117 | CTCCSJ-G-HB40478 | *Hypocrea lixii/Trichoderma harzianum* | Inner Mongolia |
| 118 | CTCCSJ-G-HB40481 | *Hypocrea lixii/Trichoderma harzianum* | Inner Mongolia |
| 119 | CTCCSJ-G-HB40482 | *Hypocrea lixii/Trichoderma harzianum* | Inner Mongolia |
| 120 | CTCCSJ-G-HB40483 | *Hypocrea lixii/Trichoderma harzianum* | Inner Mongolia |
| 121 | CTCCSJ-G-HB40484 | *Trichoderma rossicum* | Inner Mongolia |
| 122 | CTCCSJ-G-HB40485 | *Trichoderma koningiopsis* | Inner Mongolia |
| 123 | CTCCSJ-G-QT40486 | *Hypocrea atroviridis* | Inner Mongolia |
| 124 | CTCCSJ-G-HB40487 | *Hypocrea lixii/Trichoderma harzianum* | Inner Mongolia |
| 125 | CTCCSJ-G-HB40490 | *Hypocrea lixii/Trichoderma harzianum* | Inner Mongolia |
| 126 | CTCCSJ-G-HB40495 | *Trichoderma koningiopsis* | Inner Mongolia |
| 127 | CTCCSJ-G-HB40496 | *Hypocrea schweinitzii/Trichoderma citrinoviride* | Inner Mongolia |
| 128 | PDA X35-1 | *Hypocrea schweinitzii/Trichoderma citrinoviride* | Inner Mongolia |
| 129 | XZ X46-1 | *Trichoderma koningiopsis* | Inner Mongolia |
| 130 | XZ N236-1 | *Trichoderma gamsii* | Inner Mongolia |
| 131 | PDA N239-3 | *Trichoderma hamatum* | Inner Mongolia |
| 132 | XZ N71-3 | *H.schweinitzii/T. citrinoviride* | Inner Mongolia |
| 133 | XZ X171-1 | *Hypocrea lixii/Trichoderma harzianum* | Inner Mongolia |
| 134 | PDA N182-1 | *Trichoderma harzianum* | Inner Mongolia |
| 135 | CTCCSJ-G-HB40547 | *Trichoderma harzianum* | Xinjiang |
| 136 | CTCCSJ-G-HB40551 | *Trichoderma harzianum* | Xinjiang |
| 137 | CTCCSJ-G-HB40565 | *Trichoderma harzianum* | Xinjiang |
| 138 | CTCCSJ-G-HB40582 | *Trichoderma harzianum* | Xinjiang |
| 139 | CTCCSJ-G-HB40609 | *Trichoderma harzianum* | Xinjiang |
| 140 | CTCCSJ-G-HB40613 | *Trichoderma harzianum* | Xinjiang |
| 141 | CTCCSJ-G-HB40614 | *Trichoderma harzianum* | Xinjiang |
| 142 | CTCCSJ-G-HB40615 | *Trichoderma harzianum* | Xinjiang |
| 143 | CTCCSJ-G-HB40616 | *Trichoderma harzianum* | Xinjiang |
| 144 | CTCCSJ-G-HB40618 | *Trichoderma harzianum* | Xinjiang |
| 145 | CTCCSJ-G-HB40672 | *Trichoderma harzianum* | Xinjiang |
| 146 | CTCCSJ-G-HB40732 | *Trichoderma harzianum* | Xinjiang |
| 147 | CTCCSJ-G-HB40733 | *Trichoderma harzianum* | Xinjiang |
| 148 | CTCCSJ-F-KZ40809 | *Trichoderma harzianum* | Xinjiang |
| 149 | CTCCSJ-F-KY40960 | *unidentified* | Inner Mongolia |
| 150 | CTCCSJ-G-JK40974 | *Trichoderma harzianum* | Inner Mongolia |
| 151 | CTCCSJ-G-HB40989 | *Trichoderma harzianum* | Inner Mongolia |
| 152 | CTCCSJ-G-QT40994 | *Trichoderma atroviride* | Heilongjiang |

Supplementary Table 2. The comparison of homologous nucleotide sequence with ThBMV1 obtained by metagenomic sequencing

| **contig** | **Names of mycoviruses** | **Accession numbers** | **Similarity on the whole protein sequence (Coverage) by EMBOSS-NEEDLE PROGRAM** |
| --- | --- | --- | --- |
| Contig 1116 | Cryphonectria parasitica bipartite mycovirus 1 | YP007985675.1 | 65.7%% |
|  | Penicillium aurantiogriseum bipartite virus 1 | YP009182335.1 | 62.3% |
|  | Heterobasidion RNA virus 6 | ADW82833.1 | 55.6% |
| Contig 359 | Penicillium aurantiogriseum bipartite virus 1 | YP009182329.1 | 55.5% |
|  | Cryphonectria parasitica bipartite mycovirus 1 | YP007985676.1 | 56.4% |
|  | Curvularia thermal tolerance virus | YP001976145.1 | 53.0% |

Supplementary Table 3. The similarity data of RdRP protein and hypothetical protein compared with the referenced mycovirus from NCBI.

| **Proteins** | **Names of mycoviruses** | **Accession numbers** | **Similarity on the whole protein sequence (Coverage) by EMBOSS-NEEDLE PROGRAM** |
| --- | --- | --- | --- |
| The similarity data of  RdRP protein | Cryphonectria parasitica bipartite mycovirus 1 | YP007985675.1 | 65.7% |
|  | Penicillium aurantigriseum bipartite virus 1 | YP009182335.1 | 62.3% |
|  | Heterobasidion RNA virus 6 | ADW82833.1 | 55.6% |
|  | Curvularia themal tolerance virus | YP_001976144.1 | 28.7% |
| The similarity data of  hypothetical protein | Cryphonectria parasitica bipartite mycovirus 1 | YP007985676.1, | 56.4% |
|  | Penicillium aurantigriseum bipartite virus 1. | YP009182329.1 | 55.5% |
|  | Curvularia themal tolerance virus | YP001976145.1 | 53.0% |

Supplementary Table 4. The data used in the phylogenetic analysis of RdRP protein.

| **Accession Number** | **Mycovirus name** | **Classification** | **The host** | **Similarity on the whole protein sequence (Coverage) by EMBOSS-NEEDLE PROGRAM** |
| --- | --- | --- | --- | --- |
| YP_007985675.1 | Cryphonectria parasitica bipartite mycovirus 1 | unclassified mycoviruses | *Cryphonectria parasitica* | 65.7% |
| YP_009182335.1 | Penicillium aurantiogriseum bipartite virus 1 | unclassified mycoviruses | *Penicillium aurantiogriseum* | 62.3% |
| ADW82833.1 | Heterobasidion RNA virus 6 | unclassified mycoviruses | *Heterobasidion* | 55.6% |
| YP_009273017.1 | Sclerotium hydrophilum virus 1 | unclassified mycoviruses | *Sclerotium hydrophilum* | 52.7% |
| YP_003288790.1 | Fusarium graminearum dsRNA mycovirus-4 | unclassified mycoviruses | *Fusarium graminearum* | 50.4% |
| YP_009134757.1 | Rhizoctonia fumigata mycovirus | unclassified mycoviruses | *Rhizoctonia fumigata* | 46.9% |
| YP_001976144.1 | Curvularia thermal tolerance virus | unclassified mycoviruses | *Curvularia* | 28.7% |
| YP_009094186.1 | Ustilaginoidea virens RNA virus M | Unclassified victorivirus | *Ustilaginoidea virens* | 28.2% |
| YP_009052469.1 | Alternaria longipes dsRNA virus 1 | unclassified mycoviruses | *Alternaria longipes* | 32.7% |
| YP_009154711.1 | Beauveria bassiana RNA virus 1 | unclassified mycoviruses | *Beauveria bassiana* | 33.2% |
| YP_009154709.1 | Ustilaginoidea virens unassigned RNA virus HNND-1 | unclassified mycoviruses | *Ustilaginoidea virens* | 28% |
| YP_008327312.1 | Ustilaginoidea virens partitivirus 2 | unclassified *Partitiviridae* | *Ustilaginoidea virens* | 27.7% |
| CAY25801.2 | Aspergillus fumigatus partitivirus-1 | unclassified *Partitiviridae* | *Aspergillus fumigatus* | 30.7% |
| NP_624350.1 | Fusarium solani virus 1 | Gammapartitivirus, *Partitiviridae* | *Fusarium solani* | 29.0% |
| YP_009182336.1 | Penicillium aurantiogriseum partitivirus 1 | unclassified Partitiviridae | *Penicillium aurantiogriseum* | 26.0% |
| YP_001686789.1 | Botryotinia fuckeliana partitivirus 1 | unclassified Partitiviridae | *Botryotinia fuckeliana* | 25.7% |
| ADV15446.1 | Heterobasidion partitivirus 1 | Alphapartitivirus, *Partitiviridae* | *Heterobasidion* | 24.3% |
| ADV15444.1 | Heterobasidion partitivirus 5 | Alphapartitivirus, *Partitiviridae* | *Heterobasidion* | 26.4% |
| ADV15443.1 | Heterobasidion partitivirus 4 | Alphapartitivirus, *Partitiviridae* | *Heterobasidion* | 27.7% |
| ADV15441.1 | Heterobasidion partitivirus 1 | Alphapartitivirus,  *Partitiviridae* | *Heterobasidion* | 30.7% |
| YP_009508049.1 | Heterobasidion partitivirus 1 | Alphapartitivirus,  *Partitiviridae* | *Heterobasidion* | 30.7% |
| YP_009508051.1 | Heterobasidion partitivirus 12 | Alphapartitivirus,  *Partitiviridae* | *Heterobasidion* | 30.8% |
| ADV15449.1 | Heterobasidion partitivirus 1 | Alphapartitivirus,  *Partitiviridae* | *Heterobasidion* | 25.1% |
| ADV15447.1 | Heterobasidion partitivirus 1 | Alphapartitivirus,  *Partitiviridae* | *Heterobasidion* | 24.3% |
| YP_009508048.1 | Flammulina velutipes browning virus | Alphapartitivirus,  *Partitiviridae* | *Flammulina velutipes browning* | 29.2% |
| BAA09520.1 | Fusarium solani virus 1 | Gammapartitivirus, *Partitiviridae* | *Fusarium solani* | 29.0% |
| YP_052856.2 | Penicillium stoloniferum virus S | Gammapartitivirus, *Partitiviridae* | *Penicillium stoloniferum* | 29.1% |
| YP_009177606.1 | Rosellinia necatrix partitivirus 6 | Betapartitivirus, *Partitiviridae* | *Rosellinia necatrix* | 28.1% |

Supplementary Table 5. The data used in the phylogenetic analysis of hypothetical protein.

| **Accession Number** | **Mycovirus name** | **Classification** | **The host** | **Similarity on the protein sequence by EMBOSS-NEEDLE PROGRAM (Coverage)** |
| --- | --- | --- | --- | --- |
| YP_007985676.1 | Cryphonectria parasitica bipartite mycovirus 1 | unclassified mycoviruses | *Cryphonectria parasitica* | 56.4% |
| YP_009182329.1 | Penicillium aurantiogriseum bipartite virus 1 | unclassified mycoviruses | *Penicillium aurantiogriseum* | 55.5% |
| YP_001976145.1 | Curvularia thermal tolerance virus | unclassified mycoviruses | *Curvularia* | 53.0% |
| YP_009273020.1 | Sclerotium hydrophilum virus 1 | unclassified mycoviruses | *Sclerotium hydrophilum* | 41.2% |
| YP_003288791.1 | Fusarium graminearum dsRNA mycovirus-4 | unclassified mycoviruses | *Fusarium graminearum* | 41.7% |
| YP_009134758.1 | Rhizoctonia fumigata mycovirus | unclassified mycoviruses | *Rhizoctonia fumigata* | 28.9% |
| YP_009154708.1 | Ustilaginoidea virens unassigned RNA virus HNND-1 | unclassified mycoviruses | *Ustilaginoidea virens* | 21.4% |
| AKC57300.1 | Beauveria bassiana RNA virus 1 | unclassified mycoviruses | *Beauveria bassiana* | 21.9% |
| YP_009052468.1 | Alternaria longipes dsRNA virus 1 | unclassified mycoviruses | *Alternaria longipes* | 9.5% |
| AGR45852.1 | Ustilaginoidea virens partitivirus 2 | unclassified Partitiviridae | *Ustilaginoidea virens* | 13.0% |
| AGO04405.1 | Ustilaginoidea virens partitivirus | unclassified Partitiviridae | *Ustilaginoidea virens* | 15.4% |
| NP_624351.1 | Fusarium solani virus 1 | Gammapartitivirus, *Partitiviridae* | *Fusarium solani* | 11.8% |
| ALO50132.1 | Penicillium aurantiogriseum partitivirus 1 | unclassified Partitiviridae | *Penicillium aurantiogriseum* | 13.3% |
| YP_001686790.1 | Botryotinia fuckeliana partitivirus 1 | unclassified Partitiviridae | *Botryotinia fuckeliana* | 14.4% |
| CAZ61323.2 | Aspergillus fumigatus partitivirus 1 | unclassified Partitiviridae | *Aspergillus fumigatus* | 12.5% |
| ADV15442.1 | Heterobasidion partitivirus 1 | Alphapartitivirus, *Partitiviridae* | *Heterobasidion* | 6.2% |
| YP_009508052.1 | Heterobasidion partitivirus 12 | Alphapartitivirus, *Partitiviridae* | *Heterobasidion* | 10.5% |
| BAX07484.1 | Rosellinia necatrix partitivirus 3 | unclassified Partitiviridae | *Rosellinia necatrix* | 7.4% |
| NP_604476.1 | Atkinsonella hypoxylon virus | Betapartitivirus, *Partitiviridae* | *Atkinsonella hypoxylon* | 2.7% |
| YP_009508050.1 | Heterobasidion partitivirus 1 | Alphapartitivirus, *Partitiviridae* | Heterobasidion | 6.2% |
| YP_009177605.1 | Rosellinia necatrix partitivirus 6 | Betapartitivirus, *Partitiviridae* | *Rosellinia necatrix* | 5.5% |

Supplementary Table 6. The parameters of the best model for phylogenetic trees of RdRP protein, hypothetical protein and the two proteins together

| **ORF** | **Best model** | **Bayesian Information Criterion (BIC)** | **Gamma (α value)** | **Frequency of invariant sites (I)** | **rates of amino acid substitutions (R)** |
| --- | --- | --- | --- | --- | --- |
| ORF1 | LG+G+I | 10259.96184 | 1.822628291 | 0.068028818 | 0.055941 |
| ORF2 | WAG+G+I | 24718.57644 | 15.94510092 | 0.00818819 | 0.043972 |
| ORF1+ORF2 | WAG+G+I | 28536.355 | 2.787229766 | 0.028916643 | 0.043972 |

Supplementary Fig.1: The detection of dsRNA from *Trichoderma harzianum* strain 137 by the DNase Ι and S1 nuclease successively. (A) dsRNA sample was treated with DNase Ι and electrophoresed in a 1.5% agarose gel and detected on a UV transilluminator. (B) dsRNA sample was treated with S1 nuclease and electrophoresed in a 1.5% agarose gel and detected on a UV transilluminator.


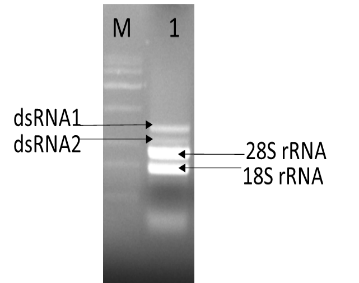

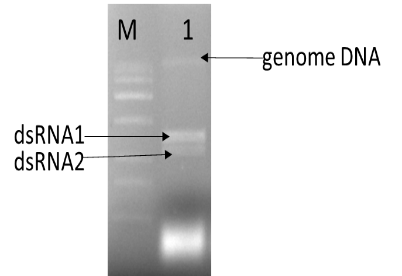


A. B.

Supplementary Fig.2: Detection of fragments of virus genomes by RT-PCR using specific primer pairs from metagenomic sequencing data. Lines 1 and line 2 were dsRNA1 segment detection. Lines 3 and 4 were dsRNA2 segment detection.


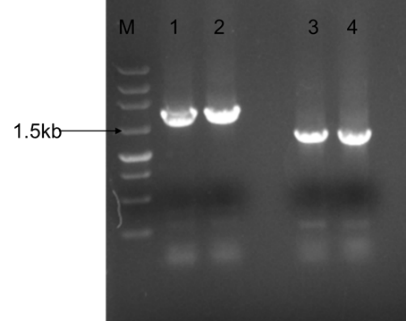


Supplementary Fig.3: Maximum likelihood phylogenetic trees inferred from the concatenated amino acid sequences of RdRP and the hypothetical protein using the WAG+G+I model of amino acid substitution. Percentages on the nodes represent statistical support based on the bootstrap method upon 1000 pseudoreplicates.


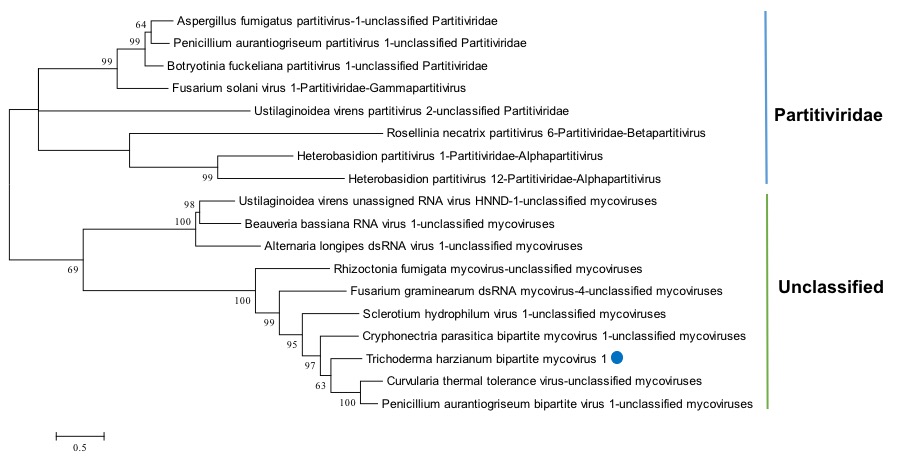

Supplement: Supplementary file 1 — Supplementary material 1 (DOCX 291 kb) [file 705_2019_4145_MOESM1_ESM.docx]
